# Childhood Exposure to War and Depressive Symptoms or a Current Major Depressive Episode in Late Adulthood

**DOI:** 10.1093/geroni/igaf122.3731

**Published:** 2025-12-31

**Authors:** Jin-kyung Lee, Gwang Suk Kim

**Affiliations:** Yonsei University College of Nursing, Yonsei University, Seoul, Republic of Korea; Yonsei University College of Nursing, Yonsei University, Seoul, Republic of Korea

## Abstract

At the end of World War II, the Korean War occurred from 1950 to 1953. During the war, people experienced unwanted separation from family members and/or leaving their hometown. It is well-known that children exposed to war have a higher risk of post-traumatic stress and depression soon after. However, little is known about its long-term effects, primarily on mental health in late adulthood. This research investigates whether the generation exposed to the war in their childhood is more likely to be vulnerable to depression in late adulthood compared to other generations. Out of 287 participants aged 55 to 89 years old, 124 older adults experienced the Korean War in their childhood. Data collected via self-report surveys and face-to-face interviews with trained researchers were analyzed by multivariate regression and logistic regression. Hamilton depression rating scale and the Mini-International Neuropsychiatric Interview were used to screen for depressive symptoms or a current major depressive episode. The results demonstrate that the older generation exposed to the Korean War in their childhood compared to others had a higher likelihood of experiencing depressive symptoms (B(SE) = -.14(.06), 95% CI = -.26∼-.01, p<.05) or a current major depressive episode (OR = .81, 95% CI = .68∼.98, p <.05) in low-stress situations. Considering the higher risk of experiencing depressive symptoms and a major depressive episode in late adulthood among the generation exposed to the war, periodic screening and targeted interventions are essential to relieve stress and improve mental health for supporting their healthier aging.

